# Despite high levels of expression in thymic epithelial cells, miR-181a1 and miR-181b1 are not required for thymic development

**DOI:** 10.1371/journal.pone.0198871

**Published:** 2018-06-27

**Authors:** Heather E. Stefanski, Yan Xing, Patricia A. Taylor, Stefano Maio, Jorge Henao-Meija, Adam Williams, Richard A. Flavell, Georg A. Hollander, Bruce R. Blazar

**Affiliations:** 1 Department of Pediatrics, University of Minnesota, Minneapolis, MN, United States of America; 2 Department of Paediatrics, University of Oxford, Oxford, United Kingdom; 3 Department of Pathology and Laboratory Medicine, Perelman School of Medicine, University of Pennsylvania, Philadelphia, PA, United States of America; 4 The Jackson Laboratory, JAX Genomic Medicine, Farmington, CT, United States of America; 5 Department of Immunobiology, Yale University, School of Medicine, New Haven, CT, United States of America; 6 Department of Biomedicine, University of Basel, and Basel University Children's Hospital, Basel, Switzerland; University of São Paulo, BRAZIL

## Abstract

MicroRNAs (miRNAs) have been shown to be key modulators of post-transcriptional gene silencing in many cellular processes. In previous studies designed to understand the role of miRNAs in thymic development, we globally deleted miRNA exclusively in thymic epithelial cells (TECs), which are critical in thymic selection. This resulted in the loss of stromal cells that instruct T cell lineage commitment and affect thymocyte positive selection, required for mature T cell development. Since murine miR-181 is expressed in the thymus and miR-181 deficiency disrupts thymocyte development, we first quantified and thereby demonstrated that miR181a1 and miR181b1 are expressed in purified TECs. By generating mice with TEC targeted loss of miR-181a1 and miR-181b1 expression, we observed that neither TEC cellularity nor thymocyte number nor differentiation was adversely affected. Thus, disrupted thymopoiesis in miR-181 deficient mice was not due to miR-181 loss of expression in TECs. Importantly, in mice with restricted TEC deficiency of miR-181a1 and miR-181b1, there were similar numbers of mature T cells in the periphery in regards to frequencies, differentiation, and function as compared to controls. Moreover miR-181a1 and miR-181b1 were not required for maintenance of thymus integrity over time, as thymic involution was not accelerated in gene-targeted mice. Taken together our data indicate that miR-181a1 and miR-181b1 are dispensable for TEC differentiation, their control of thymocyte development and mature T cell export to and homeostasis within the periphery.

## Introduction

A normal thymic stromal composition and arrangement are essential for growth, differentiation and T cell receptor repertoire selection of thymocytes. The thymus is composed of thymic epithelial cells (TECs), fibroblasts, B cells and macrophage/dendritic cells with the predominant population being thymocytes[1]. TECs form a three-dimensional matrix that reaches from the subcapsular cortical area to the core of the medulla with cortical TEC (cTEC) and medullary TEC (mTEC) subsets defining these compartments according to their functional, structural and antigenic features [2, 3]. Bidirectional interactions between developing thymocytes and the stroma are critical to maintain a normally structured and regularly functioning microenvironment able to support thymocyte development[2, 4, 5]. Developing thymocytes can be distinguished by their CD4 and CD8 cell surface expression. CD4^−^CD8^−^ (double-negative, DN) thymocytes mature to become CD4^+^CD8^+^ (double-positive, DP) thymocytes and undergo positive selection on cTECs. DP thymocytes continue their maturation into CD4^+^ or CD8^+^(single-positive, SP) thymocytes which then are subjected to negative selection first by cTECs and, then, upon migration to the medulla, by mTECs and medullary dendritic cells (DCs). mTECs can be further characterized based on their MHC II cell surface expression into largely immature and terminally differentiated mTEClo and mature mTEChi populations[6]. The mTEClo population are heterogeneous with some cells giving rise to mTEChi or to cells that express involucrin and contribute to Hassal’s corpuscles while others express CCL21 that attracts positively selected thymocytes to the medulla[6–8]. The mTEChi population is also heterogeneous, not least with regards to expression of the autoimmune regulator (AIRE)[9], critical for establishing self-tolerance of medullary thymocytes. AIRE is a transcription factor that controls the ectopic expression of a large set of peripheral tissue antigen genes in mTECs[9]. SP thymocytes complete their maturation in the medulla after which they are exported into the periphery as mature T cells.

Micro-RNAs (miRNAs) are non-protein coding molecules that downregulate transcription by degradation of target mRNAs or translation via translational repression[10]. MiRNA transcripts are first processed by the catalytic activity of Drosha, which are subsequently formed into ~22 nucleotide-double-stranded miRNAs by the enzyme Dicer[10–12]. One of the strands, called the guide strand, is loaded into the RNA-induced silencing complex (RISC)[13]. If the complex contains the endonuclease Argonaute 2 (Ago2), target mRNAs bind to Ago2 and are either degraded or translation is inhibited[13]. MiRNAs can potentially regulate a large number of genes; many miRNAs act in combination to regulate the same target genes, and predicted miRNA target genes are engaged in a wide variety of biological processes including T cell fate [11].

In order to better understand the role of miRNA in thymic development, mice were generated that lacked *dicer1* expression in TECs[14]. We found that miRNAs were critical to ensure that T cell fate, thymocyte positive selection and central tolerance induction would occur. Together, these studies demonstrated that miRNAs are essential for the maintenance of a regularly composed and correctly functioning thymic microenvironment. Macedo and colleagues discovered that AIRE can play a role as a controller of transcription of miRNAs that are located within genomic regions encompassing open reading frames and/or mRNA genes[15]. In a recent analysis of the role of miRNAs in mTEC biology in relation to promiscuous gene expression and AIRE, the investigators found that there was a mutual interdependence of miRNAs and AIRE[16]. Studies have also been performed to determine the molecular mechanism associated with loss of thymic self-tolerance utilizing autoimmune mTECs from NOD mice compared to BALB/c mTECs[17]. In this investigation, the authors found that AIRE expression was unchanged, but both AIRE-dependent and AIRE-independent peripheral tissue antigen mRNA levels were downregulated in the NOD mTECs and miRNAs were also differentially expressed[17]. Passos and colleagues found that tmiRNAs in mTECs are important in organization of the thymic architecture and also act as posttranscriptional controllers of peripheral tissue antigens[18].

The importance of miR-181 genes has been shown to be important in cellular growth, development, endothelial cell function and also plays an important role in the immune system[19] [20] [21]. The family of miR181 genes is encoded by 6 miRNAs on three separate chromosomes- MiR-181a1, miR-181a2, miR-181b1, miR-181b2, miR-181c, and miR-181d[22]. The mature forms of miR-181a1 and miR-181a2, as well as those of miR-181b1 and miR-181b2, are identical in sequence. Whereas miR-181a-1 and miR-181b-1 are on mouse chromosome 1, ~150-bp apart, miR-181a-2 and miR-181b-2 are on mouse chromosome 2, 1.1 kb apart from each other [23] MiR-181a is highly expressed in the thymus and at lower levels by cells in the heart, lymph nodes and bone marrow (BM)[11, 13]. Ectopic miR-181a expression in hematopoietic stem cells (HSCs) increases B cells and decreases CD8+ T cells, indicating that hematopoietic lineage-specific miRs can regulate immune development[19]. Moreover, increasing miR-181a expression in immature T cells reduced their sensitivity to peptide antigens and, as a result of down-regulating multiple phosphatases[19], impaired both positive and negative selection. Deletion of both miR-181 and miR-181 in mice perturbed Natural Killer T (NKT) cell and thymocyte development by regulation through PTEN phosphatase modulation of phosphatidylinositol 3-kinase (PI3K) that affects their anabolic activity[24]. These mice had a decrease in the absolute number of thymocytes further emphasizing the important role miR-181 plays in thymus[24]. To determine whether restricted deletion of miR-181a1 and miR-181b1 in TECs would affect their biology, thymocyte development or peripheral T cell homeostasis, mice were generated that lacked *Mir181a1/b1* expression exclusively in TECs.

## Materials and methods

### Mice

Mice were kept under specific pathogen-free conditions and used according to federal and institutional regulations. Mice with a conditional *Mir181a1/b1* allele (Mir181a1/b1^fl/fl^)(mirbase.org)[24] were crossed to transgenic animals expressing the Cre recombinase under the FoxN1 promoter [25] termed FoxN1-Cre::Mir181a1/b1^fl/fl^. The IACUC and IBC Committees at the University of Minnesota approved all protocols. All personnel are trained by Research Animal Resources (RAR) and need to undergo additional training by laboratory-trained personnel. There are SOPs for all mice procedures in the lab that must be followed.

### Organ isolation, flow cytometry and cell sorting

Isolation of TECs was performed as previously described[26]. Briefly, thymic lobes were digested with 0.5 mg/ml Liberase TH (Roche Diagnostics) and 100 U/ml DNaseI (Roche Diagnostics) in RMPI 1640 at 37°C for 30–60 min and mechanically disrupted. TECs were enriched to 15–20% by panning before cell sorting. The TEC-enriched cells were stained with fluorochrome-conjugated antibodies including anti-CD45 (30-F11), CD326 (G8.8), MHC II (AF6-120.1), Ly-51 (6C3) and Ulex Europaeus Agglutinin I (UEA-I). TEC subpopulations were sorted on a FACSAria (Becton Dickinson) and purities were over 90%.

Splenocytes, thymocytes and LNs were suspended in 2% fetal calf serum/phosphate-buffered saline (PBS), and 10^6^ cells were incubated with appropriate fluorochrome-conjugated monoclonal antibodies (BD Pharmingen, San Jose, CA) for 30 minutes at 4°C. A total of 10^5^ live events were acquired on a Fortessa flow cytometer (BD Pharmingen) and analyzed with FlowJo software (TreeStar, San Jose, CA). The antibodies used were CD3 (KT-3), CD4 (GK1.5; BioLegend), CD8 (53–67; eBioscience), CD19 (ID3), CD44 (IM7; eBioscience), CD62L (MEL-14; BD Biosciences) and NK1.1 For TEC analysis, thymic lobes were cut into small pieces and then incubated at 37°C for 60 min in HBSS containing 2% (w/v) FCS (Perbio), 100 μg/ml Collagenase/Dispase (Roche Diagnostics), and 40 ng/ml DNaseI (Roche). TECs were enriched using AutoMACS (Miltenyi Biotec) and stained for the expression of epithelial cell adhesion molecule (EpCAM) (G8.8, DSHB; University of Iowa), CD45 (30F11; eBioscience), MHC class I (MHC I; AF6-88.5; BioLegend), MHC class II (MHC II; AF6-120.1; BioLegend), Ly51 (6C3; BioLegend), Dll-4 (gift from Robson MacDonald, University of Lausanne, Lausanne, Switzerland), and UEA-1 (Reactolab). Flow cytometric analysis and cell sorting were performed (FACSAria) using FACSFortessa (BD Biosciences) and FlowJo software (Tree Star).

### miRNA detection and gene expression profiling

RNA was isolated from sorted separated thymocytes (controls), cTECs and mTECs and reverse transcripted using the QIAGEN miRNeasy Micro Kit (QIAGEN) and Taqman MicroRNA Reverse Transcription Kit (ThermoFisher Scientific), respectively, according to the manufacturer’s instructions. Reverse transcription polymerase chain reactions (RT-PCR) were conducted using TaqMan Universal Master Mix II, no UNG (ThermoFisher Scientific).

All quantitative PCR (qPCR) reactions were performed using a StepOnePlus Real Time PCR System (Applied Biosystems) with each sample being analyzed in triplicate from 3 biologic experiments. miRNA181^a/b^ relative expression was calculated as 2^-∆Ct^ values. Error bars represent standard deviation (SD), and statistical significance was calculated using a one-tailed, unpaired t-test. p<0.05 was considered to be significant. TaqMan Probes used for miRNA181 detection were: mmu-miR-181a (mature miRNA sequence): ACCGACCGUUGACUGUACCUUG, miRBase Accession Number MIMAT0005443. mmu-miR-181b (mature miRNA sequence): CUCACUGAACAAUGAAUGC, miRBase Accession Number MIMAT0017067.

## Statistical analysis

Statistical analysis was performed using Student *t* test (unpaired, two-tailed) using the Graph Pad Prism software (version 6). Probability values were classified into four categories: *p* > 0.05 (NS), *0.05 ≥ *p* > 0.01, **0.01 ≥ *p* > 0.001, and ***p ≤ 0.0001.

## Results

### MiR-181a1 and MiR-181b1 expression is found in TECS

**Fig 1:**
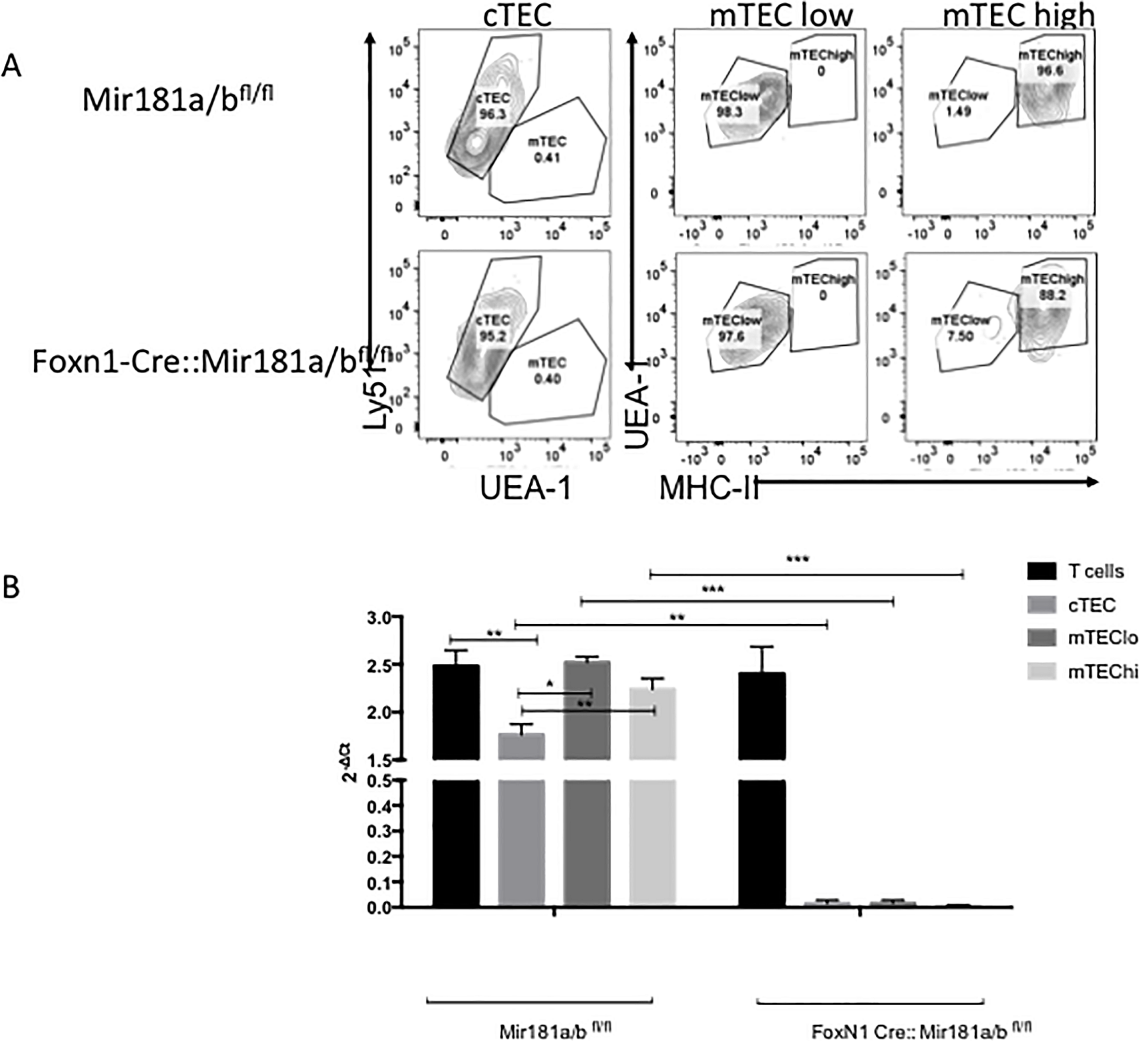
miR-181a1 and miR-181b1 expression in thymocytes, cTEC and mTECs in Mir181a1/b1^fl/fl^ and FoxN1-Cre::Mir181a1/b1^fl/fl^ mice. Thymocytes, cTECs or mTECs were isolated using flow cytometry by staining with CD45, EpCAM, Ly51, UEA-1 and MHC II. Fig 1A. Thymocytes (CD45+) were sorted using a FacsAria. TECS (CD45-,EpCAM+) were isolated and then sorted into subpopulations: cTEC (UEA^−^1-Ly51^+^), mTEClo (UEA-1^+^Ly51^−^) mTEChi(UEA-1^+^Ly51^-^I-A^b^) from both Mir181a1/b1^fl/fl^ mice and Foxn1-Cre::Mir181a1/b1^fl/fl^. RNA was isolated and miR-181a1 and miR-181b1 was quantified using RT-PCR in the different populations. * = P<0.05, ** = P<0.01, *** = P<0.001.

It has been well defined that miR-181a is highly expressed in the thymus, especially in DP thymocytes[19]. We first determined whether miR-181a1 and miR-181b1 also were expressed in TECs. For this purpose, wild type TECs were isolated and separated by flow cytometry sorting into cTEC (CD45-EPCAM+LY51+), mTEChi (CD45-EPCAM+UEA1+I-A^b^hi) and mTEClo (CD45-EPCAM+,UEA1+I-A^b^lo) populations based on CD45, EPCAM, UEA1, Ly51 and I-A^b^ expression (Fig 1A). Quantitative RT-PCR was performed to determine the expression level of miR-181a1 and miR-181b1. Unseparated thymocytes, cTECs and mTECs expressed miR-181a1 and miR-181b1 at detectable copy numbers, albeit with significantly lower levels in cTECs (Fig 1B). Taken together these data demonstrated that *miR-181a1* and *miR-181b1* are expressed in all TEC subsets providing an opportunity for their influence on TEC biology.

### MiR-181a1 and MiR-181b1 expression in TECs from FoxN1-Cre::Mir181a1/b1^fl/fl^

With evidence of miR-181a1 and miR-181b1 expression in TECs, we next generated mice that lack the expression of miR-181a1 and miR-181b1 in TECs to interrogate their role in TEC development and function. To ensure a TEC-restricted deletion of both miRs, mice with a conditional *Mir181a1/b1* allele (Mir181a1/b1^fl/fl^) were crossed to animals that express the Cre recombinase under the transcriptional control of the *FoxN1* locus, expressed in TECs and skin epithelial cells. Due to the close physical proximity of *miR-181a1* and *miR-181b1* and their very low expected frequency of gene cross-over, mice individually deficient for one or the other of these two miRNA could not be generated to assess the individual roles of miR-181a1 and miR-181b1 in TEC development. [23][27]. Mir181a1/b1^fl/fl^ mice were used as controls and compared to heterozygous Cre-transgenic Mir181a1/b1^fl/fl^ mice (designated Foxn1-Cre:: Mir181a1/b1^fl/fl^).

To confirm that miR-181a1 and miR-181b1 expression was undetectable in TECs from FoxN1-Cre::Mir181a1/b1^fl/fl^ crosses, TECs were isolated by flow cytometry sorting into cTEC, mTEChi and mTEClo (see Fig 1A). As shown in Fig 1B, *miR-181a1* and *miR-181b1* expression was undetectable in TECs but discernable in thymocytes.

### TEC differentiation and cellularity are independent of *mir181a1* and *miR-181b1* expression

**Fig 2:**
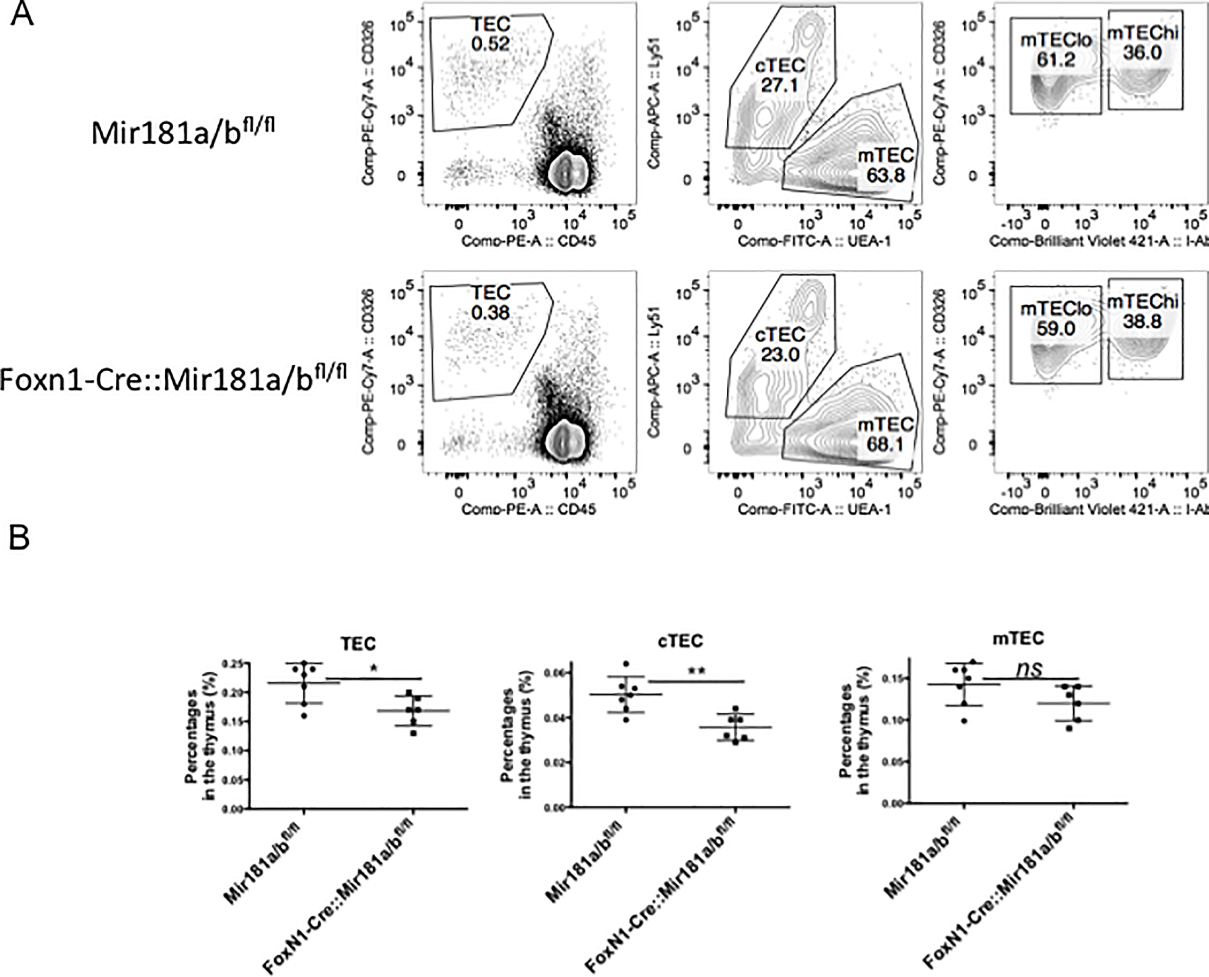
The effects of miR-181a1 and miR-181b1 loss on TEC distribution. Fig 2A. Flow cytometric analysis of TEC (CD45^−^EpCAM^+^) subpopulations isolated in mice. The relative frequency of cTEC (UEA^−^1-Ly51^+^), mTEClo (UEA-1^+^Ly51^−^) mTEChi(UEA-1^+^Ly51^+^I-A^b+^) are shown in both Mir181a1/b1^fl/fl^ mice compared to Foxn1-Cre::Mir181a1/b1^fl/fl^. These are representative dot plots. This experiment was performed twice with at least 3 mice per group. Fig 2B. Flow cytometric analysis of TEC (CD45^−^EpCAM^+^) subpopulations isolated in mice. The relative frequency of cTEC (UEA^−^1-Ly51^+^), mTEClo (UEA-1^+^Ly51^−^) mTEChi(UEA-1^+^Ly51^+^I-A^b+^) are shown as a percentage in the thymus in both Mir181a1/b1^fl/fl^ mice compared to Foxn1-Cre::Mir181a1/b1^fl/fl^. This experiment was performed twice with at least 3 mice per group. Unpaired non-parametric t test was performed. * = P<0.05, ** = P<0.01.

We next probed for the biological consequences of a loss miR-181a1 and miR-181b1 in TEC differentiation. Fig 2A shows representative plots from both Mir181a1/b1^fl/fl^ and Foxn1-Cre::Mir181a1/b1^fl/fl^ mice. Interestingly there was a significant difference in the percentage of total TECs in the Mir181a1/b1^fl/fl^ compared to Foxn1-Cre::Mir181a1/b1^fl/fl^ mice (0.23% compared to 0.17% respectively, p = 0.0309) as shown in Fig 2B. This difference was due to a decreased percentage of cTECS in Foxn1-Cre::Mir181a1/b1^fl/fl^ mice and not mTECs (cTEC: Mir181a1/b1^fl/fl^ = 0.05% and Foxn1-Cre::Mir181a1/b1^fl/fl^ mice = 0.0355%; p = 0.0058; mTEC: percentage of the total thymus was not significant between groups).

**Fig 3:**
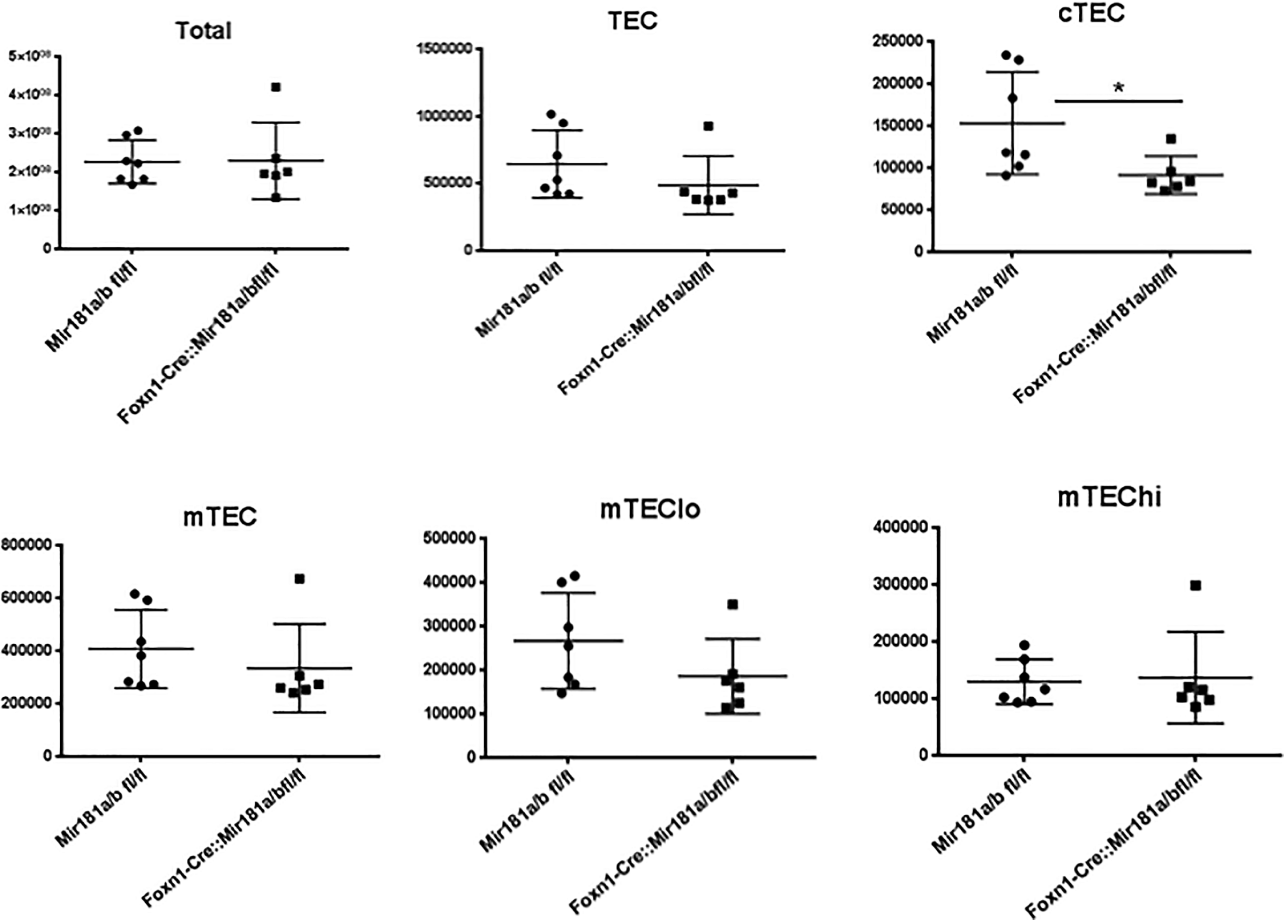
miR-181a1 and miR-181b1 does not affect TECs based on absolute numbers. A. Flow cytometric analysis of TEC (CD45^−^EpCAM^+^MHC II^+^) subpopulations isolated in mice. The absolute number of total thymocytes, TEC, cTEC (UEA^−^1-Ly51^+^), mTEC (UEA1+),mTEClo (UEA-1^+^Ly51^−^), mTEChi(UEA-1^+^Ly51^-^MCHII^+^) is shown in both Mir181a1/b1^fl/fl^ mice compared to Foxn1-Cre::Mir181a1/b1^fl/fl^. This experiment was done two times with at least 3 mice per experiment.

We next quantified thymocytes and TEC subsets in Foxn1-Cre::Mir181a1/b1^fl/fl^ mice and controls. As shown in Fig 3, both mouse strains of mice had comparable numbers of thymocytes, total TECs, mTECs, mTEChi and mTEClo cells, however cTECs were significantly lower in Foxn1-Cre::Mir181a1/b1^fl/fl^ mice. Taken together, the loss of *miR-181a1* and *miR-181b1* expression in TECs had a small effect on the total number of cTECs, but despite this, thymocyte cellularity and total number of TECs were not impacted in thymii from Foxn1-Cre::Mir181a1/b1^fl/fl^ mice.

### Thymocyte development does not require expression of MiR-181a1 and MiR-181b1 in TECs

**Fig 4:**
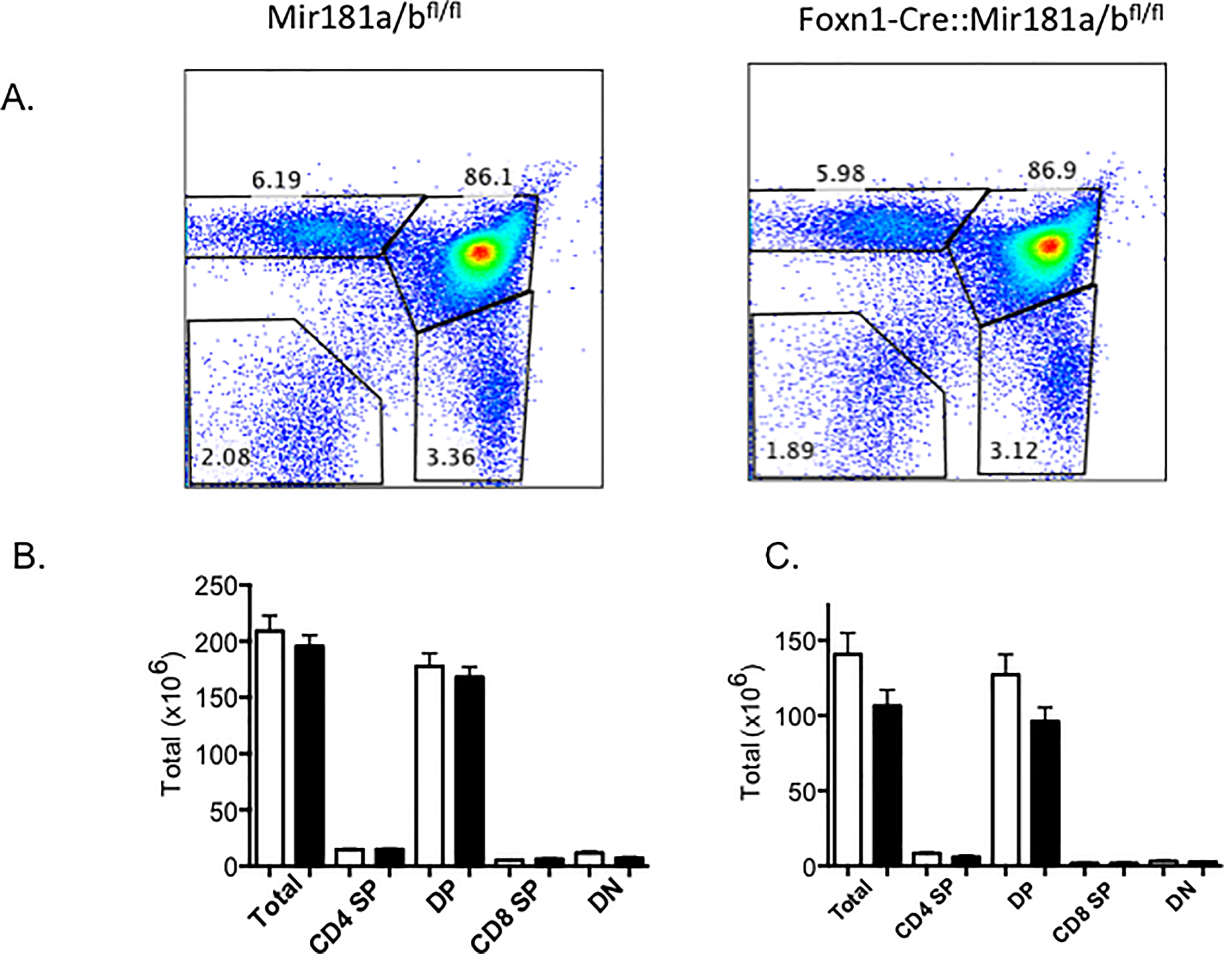
Thymic profiles and numbers of cells are not altered due to loss of miR-181a1 and miR-181b1 in TECs. Flow cytometric analysis for the cell-surface expression of CD4 and CD8 on thymocytes isolated from Mir181a1/b1^fl/fl^ and Foxn1-Cre::Mir181a1/b1^fl/fl^ mice. Numbers denote the percentage of cells within the given gates in a representative experiment. B. Thymocytes were harvested at two different time points 6 weeks (B) and 4 months (C). Absolute numbers are shown of the thymic subsets. There were no significant differences between Mir181a1/b1^fl/fl^ (white) and Foxn1-Cre::Mir181a1/b1^fl/fl^ mice (filled).

We next assessed CD4 and CD8 expression profiles of Mir181a1/b1^fl/fl^ and Foxn1-Cre::Mir181a1/b1^fl/fl^ mice. As shown in Fig 4A, the pattern observed was identical for both mouse strains revealing normal frequencies of all discernable stages using this analysis. Moreover, normal thymopoiesis remained unchanged with older age since the distribution of thymocyte subsets were identical at both 6 weeks and 4 months of age for Foxn1-Cre::Mir181a1/b1^fl/fl^ and Mir181a1/b1^fl/fl^ mice (Fig 4B and 4C).

### MiR-181a1 and MiR-181b1 expression does not skew T cell populations in the spleen and lymph nodes.

**Fig 5:**
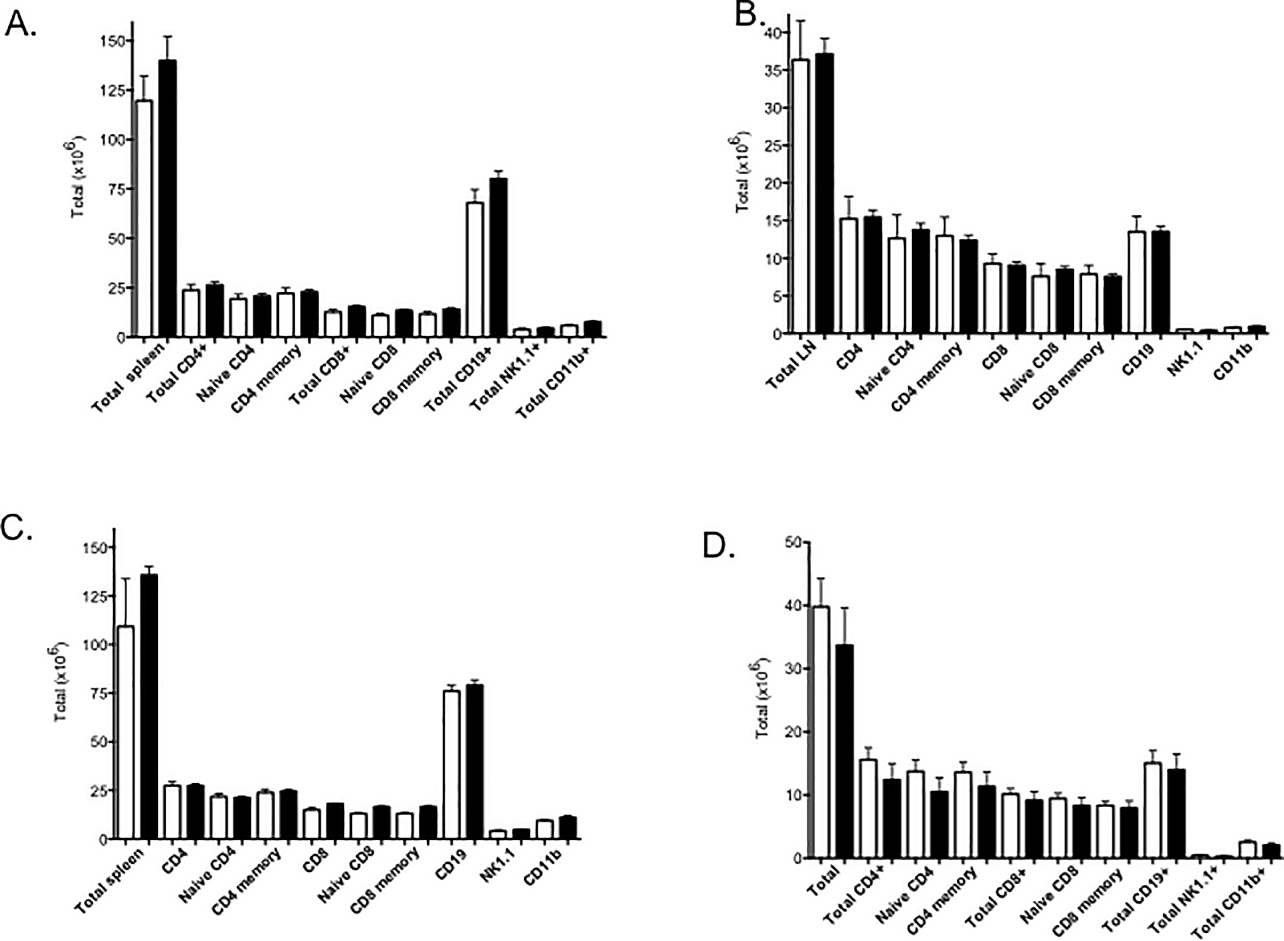
Peripheral cell compartments are not affected by loss of miR-181a1 and miR-181b1 in TECs. Spleen (A,C) and lymph nodes (B, D) were harvested at 6 weeks (A,B) and at 4 months (C,D). Absolute numbers are shown of the different subsets. There were no significant differences between Mir181a1/b1^fl/fl^ (white) and Foxn1-Cre::Mir181a1/b1^fl/fl^ mice (filled). This experiment was repeated twice with at least 5 mice in each group.

Although there were no differences in the thymic compartment in Foxn1-Cre::Mir181a1/b1^fl/fl^ mice, the peripheral compartment is essential for a productive T cell response to foreign antigens and pathogens, and process such as tolerance to endogenous autoantigens and immune surveillance to tumor antigens. Because TECs play an important role in negative selection, the lack of miR-181a1 and miR-181b1 could have profound effects on the mature T cell repertoire. As shown in Fig 5, the cellularity was similar in regards to the absolute cell numbers of T cells, B cells, DCs, or NK cells from spleens (Fig 5A and 5C) lymph nodes (Fig 5B and 5D) both from young (Fig 5A and 5B) or older (Fig 5C and 5D) Foxn1-Cre::Mir181a1/b1^fl/fl^ as compared to Mir181a1/b1^fl/fl^ mice. Moreover, peripheral T cells selected by TEC deficient in miR-181a1 and miR-181b1 expression proliferated normally in response to anti-CD3 and IL-2 (data not shown). Hence, Foxn1-Cre::Mir181a1/b1^fl/fl^ mice had peripheral T cells capable of responding to T cell receptor associated signals.

## Discussion

MiR-181a has been shown to be expressed at high levels in the thymus[19, 21]. Our studies addressed the role of miR-181a1 and miR-181b1 in TECs and the effects on TEC development. In all subsets of TECs, expression of miR-181a1 and miR-181b1 was present at levels equal to thymocytes. Interestingly, there was a decrease in the percentage of total TECs in thymii from Foxn1-Cre::Mir181a1/b1^fl/fl^ mice, which was due to a decrease in the cTEC compartment. Although this did not result in a decrease an absolute number of total TECS, but did result in a lower number of cTECS. Despite this difference, positive selection was not affected in these mice.

Li et al.[19] showed that miR-181a is expressed at high levels during each of the first 3 DN stages of early thymocyte development as well as in cells undergoing positive selection. Ectopic expression of miR-181a in DN thymocytes resulted in a quantitative increase in the percentage of DP cells and a decrease in the proportion of CD8+ SP cells, suggesting that miR-181a influences early thymic development at pre-TCR and TCR-dependent stages. Moreover, transgenic miR-181a expression augmented TCR-mediated T cell activation following antigenic stimulation. In mature CD8+ T cells that were transduced with miR-181a, the TCR was much more sensitive to antigenic stimulation based on the number of peptides required to produce IL-2. Studies by Chen et al.[21], evaluated the ectopic expression of miR-181 in murine lineage negative bone marrow cells. This resulted in decreased number of T cells and an increase number of B cells showing that miR-181 acts as a positive regulator of B cells. In mice with a miR-181 deletion, the absolute number of thymocytes is significantly decreased, highlighting the important role miR-181 plays in thymus[24]. Our data showed that cTECS and thymocytes express similar levels of both miR-181a1 and miR-181b1 (Fig 1B). Although deletion of miR-181a1 and miR-181b1 in cTECs impacted their absolute number, it did not affect their the cTECs’ capacity to positively select developing thymocytes.

Previous studies have shown that MiR-181a-5p is expressed in TECs at high levels in young mice (1 month of age) but decreased significantly with age[28]. The thymus undergoes involution during the aging process which reduces naïve T cell output and increases self-reactive T cells [29]. In order to determine the functional role of miR-181-5p in TEC, the cell line mTEC1 was transfected with miR-181a-5p mimic, miR-181a-5p inhibitor, or a negative control and their *in vitro* proliferation was quantified[28]. A direct correlation was observed between high miR-181a-5p levels and proliferation possibly mediated by Smad3 phosphorylation and blocked activation of signaling by transforming growth factor-beta, a negative regulator of proliferation. Contrary to an over-expression of *miR-181a* and *miR-181b* in TEC, *miR-181a1* and *miR-181b1* deletion in Foxn1-Cre::Mir181a1/b1^fl/fl^ mice did not impact on total or lineage-specific TEC cellularity. Because levels of miR-181a-5p were at low levels in aged mice, one might have predicted that Foxn1-Cre::Mir181a1/b1^fl/fl^ mice would have had an involuted thymus. However, our data showed that *miR-181a1* and *miR-181b1* deletion did not affect thymic involution, suggesting that there are additional key players of this complicated process. Although miR-181a expression is a critical modulator in thymocytes, our data do not support a role for either miR-181a1 or miR-181b1 expression in TECs as deletion of these miRs had no effect on thymic development.

In summary, our data show that the targeted loss of *miR-181a1* and *miR-181b1* in vivo affected cTEC number and differentiation only, which did not impact the number or differentiation of thymocytes. We found that miR-181a1 and miR-181b1 loss does not expedite thymic involution and are not required for thymic preservation. Recently, Park *et al*. generated 46 germline-transmitted miRNA knockout mice to assess their roles in vivo and found that many miRNA knockout mice tested were viable, supporting a mechanism by which miRNAs act redundantly with other miRNAs or other pathways[30]. Taken together our data suggest that *miR-181a1* and *miR-181b1* are dispensable for TEC support of thymic development and mature T cell population in the periphery.
